# Rice Routes of Countering *Xanthomonas oryzae*

**DOI:** 10.3390/ijms19103008

**Published:** 2018-10-02

**Authors:** Zhiyuan Ji, Chunlian Wang, Kaijun Zhao

**Affiliations:** National Key Facility for Crop Gene Resources and Genetic Improvement (NFCRI), Institute of Crop Sciences, Chinese Academy of Agriculture Sciences (CAAS), Beijing 100081, China; jizhiyuan@caas.cn (Z.J.); wangchunlian@caas.cn (C.W.)

**Keywords:** rice, *Xanthomonas oryzae*, *R* genes, TALE, *Xa1*, iTALE

## Abstract

Bacterial blight (BB) and bacterial leaf streak (BLS), caused by *Xanthomonas oryzae* pv. *oryzae* and *Xanthomonas oryzae* pv. *oryzicola*, respectively, are two devastating diseases in rice planting areas worldwide. It has been proven that adoption of rice resistance is the most effective, economic, and environment-friendly strategy to avoid yield loss caused by BB and BLS. As a model system for plant—pathogen interaction, the rice—*X. oryzae* pathosystem has been intensively investigated in the past decade. Abundant studies have shown that the resistance and susceptibility of rice to *X. oryzae* is determined by molecular interactions between rice genes or their products and various pathogen effectors. In this review, we briefly overviewed the literature regarding the diverse interactions, focusing on recent advances in uncovering mechanisms of rice resistance and *X. oryzae* virulence. Our analysis and discussions will not only be helpful for getting a better understanding of coevolution of the rice innate immunity and *X. oryzae* virulence, but it will also provide new insights for application of plant *R* genes in crop breeding.

## 1. Introduction

Phytopathogenic bacteria of the *Xanthomonas* genus are causative agents of numerous destructive diseases in more than 300 plant species, including some important crops like rice, citrus, and cotton [1,2]. *X. oryzae* pv. *oryzae* (*Xoo*) and *X. oryzae* pv. *oryzicola* (*Xoc*), two close pathovars of *X. oryzae* species, cause bacterial blight (BB) and bacterial leaf streak (BLS), respectively, in rice. These are among the major diseases in most rice planting areas worldwide, leading to reduced yield and poor quality of the affected plants [3]. Unlike nonpathogenic bacteria, *Xanthomonas* species attack plants by mainly relying on various effectors secreted through different types of protein secretion systems, particularly the type III secretion system (T3S), to suppress host immunity and obtain nutrients from plants. In *X. oryzae*, T3S effectors (T3SEs) are grouped into two types: transcription activator-like effectors (TALEs) and non-TALEs (also called *Xanthomonas* outer proteins, Xops) [2,4].

TALEs, formerly cited as AvrBs3/PthA proteins, are important virulence factors of most species in the *Xanthomonas* genus. In the past few years, tremendous research progresses have been achieved in TALE biology, particularly on TALE’s virulence contribution [5,6,7,8]. The TALE proteins are injected into plant cell, then enter the nucleus, playing roles as transcription factors by activating expression of target genes to promote susceptibility or suppress effector-triggered immunity (ETI) of plants [1,9,10]. For example, PthA from *X. citri* controls pustule formation and bacterial population density in plant leaf during citrus bacterial canker by activating *CsLOB1*, a member of the Lateral Organ Boundaries gene family [11]. Similarly, AvrBs3 from *X. campestris* pv. *vesicatoria* causes hypertrophy and facilitate bacterial spreading in pepper by activating the bHLH transcription factor *UPA20* [12]. Major virulence TALEs (e.g., PthXo1 and AvrXa7) from *Xoo* activates the expression of multiple rice *SWEET* genes (e.g., *OsSWEET11* and *OsSWEET14*) to promote susceptibility [13,14,15], and Tal7 from *Xoc* activates the expression of *Os09g29100* to suppress *avrXa7*-induced resistance in rice [16]. Both *Xoo* and *Xoc* harbor large TALE repertoires, usually exceeding 8 in *Xoo* isolates and 20 in *Xoc* isolates, are significantly different from other pathovars in *Xanthomonas* [17,18,19].

Xops of *Xanthomonas* constitute another important group of virulence factors, which is mainly involved in manipulation of signaling in plant defense response. Several dozen Xops have been intensively studied. XopY_MAFF311018_ from the *Xoo* strain T7174 interacts with two receptor-like cytoplasmic kinases (RLCKs)—OsRLCK55 and OsRLCK185—and suppresses OsRLCK185 phosphorylation to interfere mitogen-activated protein kinase (MAPK) signaling [20]. XopP_MAFF311018_ targets ubiquitin E3 ligase, OsPUB44, and reduces ligase activity to suppress the PAMPs-triggered immunity (PTI) in rice [21]. It has also been shown that XopN_KXO85_, an important effector of *Xoo* strain KXO85, targets OsVOZ2 and OsXNP and utilizes OsVOZ2 for successful infection and increased virulence [22]. Additionally, XopZ_PXO99_, XopF_ITCCBB0002_, XopR_MAFF311018/1375_, and XopQ_BXO43_ have all been reported to affect plant defense and significantly contribute to strain virulence [23,24,25,26,27]. Among the 23 investigated *Xop* genes in *Xoc*, *avrBs2_RS105_* has been demonstrated to be required for full virulence of *Xoc* strains, while the *xopAA_RS105_* deletion enhanced strain virulence in rice JG30 [28]. It has been reported that the same Xop protein may contribute differently to virulence due to either different genetic backgrounds of strains or rice cultivars used for virulence assays.

The interaction between effectors and their targets is a cruel struggle between pathogen and host at molecular level. Rice–*X. oryzae* pathosystem is an excellent model for understanding effector biology, plant innate immunity, and other aspects of host–parasite interactions. In a long-time coevolution with pathogen, rice has evolved successful defense systems with the core of *R* genes to resist disease. The resistance strategies revealed so far of *R* genes adopted in rice–*X. oryzae* interactions mostly fit within two mechanisms: activating host innate immunity upon perception of the pathogen effectors and abolishing the host susceptibility through loss of interaction with effectors [29]. Interestingly, eight (*Xa1*, *xa5*, *Xa10*, *xa13*, *Xa23*, *xa25*, *Xa27*, *xa41*) out of 11 major rice resistance (*R*) genes cloned so far against *X. oryzae* mediate resistance associated with the TALEs in the course of rice–*Xanthomonas* interaction, which are grouped into three types of *R* genes, i.e., recessive, dominant nontranscriptional, and dominant TALE-dependent transcriptional resistance genes [30,31,32]. The other three cloned BB *R* genes are *Xa3*/*Xa26*, *Xa4*, and *Xa21*, which encode two types of receptor kinase proteins [33,34,35]. This review will focus on the diversified interactions between the major *R* genes or their products and the avirulence (Avr) proteins or virulent effectors in the rice–*X. oryzae* pathosystem to provide an overview of coevolution of rice innate immunity and the *X. oryzae* virulence.

## 2. Broad-Spectrum Resistance Mediated by Kinases

Plants have a precise immune system that is able to detect invading microbes and trigger a defense response [36]. The first line of innate immunity in plants, defined as PTI, is primarily made up of cell surface-localized pattern recognition receptors (PRRs), which can recognize a wide range of microbe- or pathogen-derived molecules, the so-called pathogen-associated molecular patterns (PAMPs), delivered into plant apoplast. This perception between PRRs and PAMPs triggers plant immune response (PTI) to resist a vast majority of microbes in the environment [36]. Receptor kinases (RKs) are the major components of PRRs and can alarm initial attacking from pathogens. For example, when rice is attacked by the fungus *Magnaporthe oryzae*, the host PRR OsCEBiP can recognize and bind the chitin from the fungus, forming a receptor complex with receptor-like kinases OsCERK1, and transduces signals to downstream proteins OsRacGEF1 and OsRac1 for innate immune responses [37,38].

**Figure 1:**
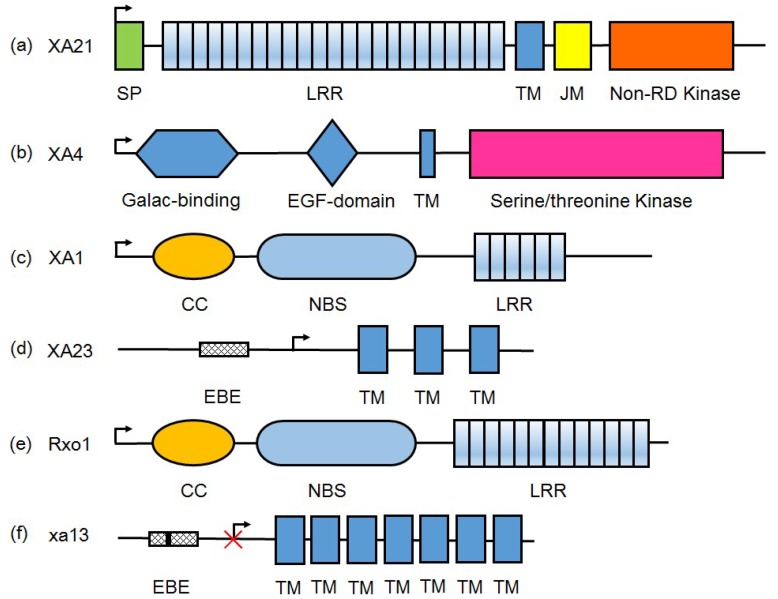
The protein domain structures of representative *R*-gene products for *Xoo* resistance. (**a**) Receptor kinase, including XA21 and XA3/XA26. XA21 is exampled for illustrations here; (**b**) Wall-associated kinase, XA4; (**c**) Nucleotide-binding leucine-rich repeat (NLR)-type, XA1; (**d**) Executor R proteins, including Xa10, XA23, and XA27. Xa23 is exampled; (**e**) Nonhost R protein from maize, Rxo1; (**f**) SWEET protein encoded by recessive type *R* genes such as *xa13*, *xa25*, and *xa41*. These recessive type *R* genes are evolved by mutation (black vertical line in the EBE box) in promoter to block transcription activator-like effectors (TALE)-induced susceptibility. xa13 is exampled here. SP: signal peptide; LRR: leucine-rich repeat domain; TM: transmembrane domain; JM: juxtamembrane domain; non-RD: non-arginine-aspartate domain; Galac-binding: galacturonan-binding domain; EGF: calcium-binding epidermal growth factor domain; CC: coiled-coil domain; NBS: nucleotide binding site; EBE: effector binding element. Arrow indicates the direction of transcription.

**Figure 2:**
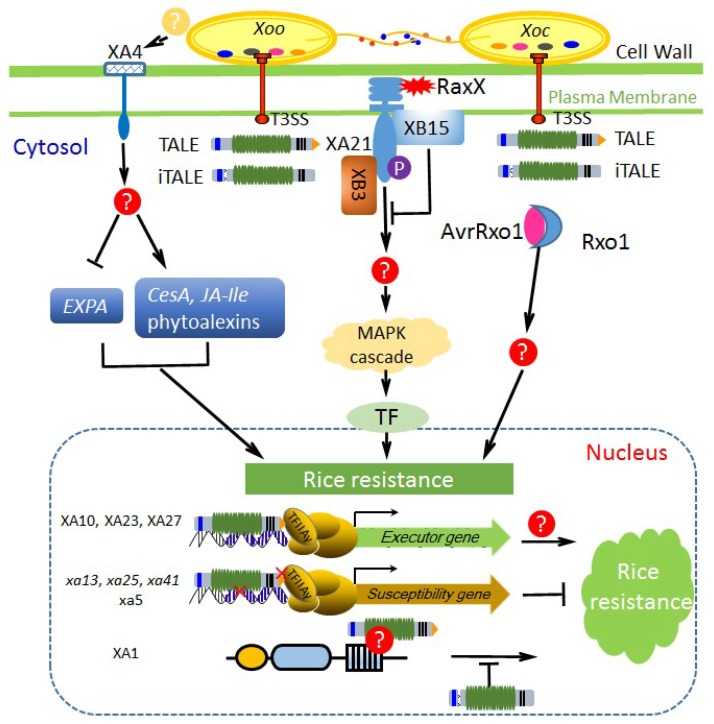
The known pathways of rice major resistance against *X*. *oryzae*. *X*. *oryzae* secretes virulence factors (multicolor oval) via protein secretion systems (e.g., T3SS) to benefit the pathogens, but some were recognized by *R* gene products in rice to trigger innate immunity. XA21 localized at the apoplast recognizes tyrosine-sulfated protein RaxX (red sharp shape) from *X*. *oryzae* and activates defense response by phosphorylating downstream targets. XB3, a ring figure ubiquitin ligase, is required for effective XA21-mediated resistance and may participate in activating the MAPK (mitogen-activated protein kinase) cascade. XB15, a PP2C phosphatase, dephosphorylates autophosphorylated XA21 and negatively regulates the resistance. XA4 is a cell wall-associated kinase and can be activated by ligands from *Xoo*, leading to induction of *CesAs* expression, inhibition of *EXPA* expression, and accumulation of JA-Ile and phytoalexins. AvrRxo1 (pink oval) is a *Xoc*-specific effector and can interact with a maize NLR protein Rxo1 (light blue crescent), resulting in hypersensitive response (HR) induction in rice. Executor *R* genes, recessive *R* genes, and *Xa1*-like NLR genes evolved in rice represent different strategies of countering TALE attacks. iTALEs from *X*. *oryzae* are suppressors of *Xa1*-mediated resistance. Question marks (?) in red and yellow backgrounds indicate the unknown interactors from rice and *X*. *oryzae*, respectively.

The rice genome encodes more than 290 leucine-rich repeat (LRR) receptor kinases [39], and the well-characterized members—*Xa21* and *Xa3*/*Xa26*—are representatives of non-RD (non-arginine-aspartate) RKs; both confer broad-spectrum resistance to *Xoo* strains. *Xa21*, originating from *Oryza longistaminata*, is the first cloned BB resistance gene and encodes a plasma membrane-localized receptor with extracellular LRR, transmembrane, juxtamembrane (JM), and cytoplasmic kinase domains (Figure 1) [35]. The function of this receptor has been intensively studied (Figure 2), and it is now clear that the JM domain plays an important role in the XA21 signal transduction pathway. In the absence of an Avr protein, XB24 (XA21 binding protein 24) binds XA21 and promotes phosphorylation of Ser/Thr sites (Ser686, Thr688, and Ser689) in the JM region through its ATPase activity, keeping the XA21 in an inactive state [40]. However, when rice plants face a challenge from *Xoo*, XA21 can recognize a sulfated Type-I-secreted protein RaxX, which is the Avr protein highly conserved in many *Xanthomonas* species [41]. Subsequently, XA21 dissociates from XB24 and excites the downstream defense response, e.g., XA21 transphosphorylates XB3, a ring figure ubiquitin ligase, to activate MAPK cascade. Furthermore, once the signal has been replayed, XA21 binds the XB15 phosphates, which attenuates the immune response, likely by dephosphorylation of amino acids required for XA21 function to negatively regulate the *Xa21*-mediated immune responses (Figure 2). RaxX is a 60-amino acid peptide similar to a PSY (plant peptide containing sulfated tyrosine) family peptide PSY1 in structure, suggesting that RaxX might have evolved to mimic plant hormone to regulate host biological process, thereby facilitating pathogen infection in the struggle [42]. Compared to *Xoo*, the RaxX homolog in *Xoc* is closer to another PSY peptide in rice OsPSY2 based on the three residues required for XA21 recognition.

*Xa3*/*Xa26*, another RK-type *R* gene, encodes a protein similar to XA21 in structure, mediating a different spectrum but also broad and durable resistance to *Xoo* strains [33,43,44,45]. A gene-dosage effect exists in *Xa3*/*Xa26*-mediated resistance—the higher expression level of *Xa3*/*Xa26* confers a stronger, wider spectrum, and whole-growth-stage resistance. The *Xa3*/*Xa26*-mediated resistance is also influenced by rice genetic backgrounds: *Oryza japonica* background and *WRKY45-1* allele are more appropriate for *Xa3*/*Xa26* expression compared to *Oryza indica* background and *WRKY45-2* allele [46].

The BB resistance gene *Xa4* has been widely utilized in developing resistant varieties since 1970s [34]. It locates closely to the *Xa3*/*X26* locus, encoding a cell wall-associated kinase (WAK) (Figure 1) and confers durable resistance to multiple *Xoo* races (e.g., Philippine race 1, 4, 5, 7, 8). Expression of *Xa4* is rapidly and strongly induced in incompatible rice–*Xoo* interactions. In *Xa4*-carrying rice, expressions of cellulose synthase (CESA) family genes are enhanced, while expression of the α-expansin (EXPA) genes is suppressed. This regulation of multiple gene expression patterns enhances the mechanical strength of rice cell wall, as well as resistance against different biotic stress, and reduces plant height. Furthermore, expression induction of *Xa4* in incompatible interactions may strengthen *CesAs* induction to reinforce the cell wall, exhibiting race-specific resistance (Figure 2). The *Xa4*-mediated resistance has been reported to be associated with increased accumulation of JA-Ile, sakuranetin, and momilactone A (Figure 2) [34].

## 3. Broad-Spectrum Resistance Mediated by the *Xa1* Family Genes

**Figure 3:**
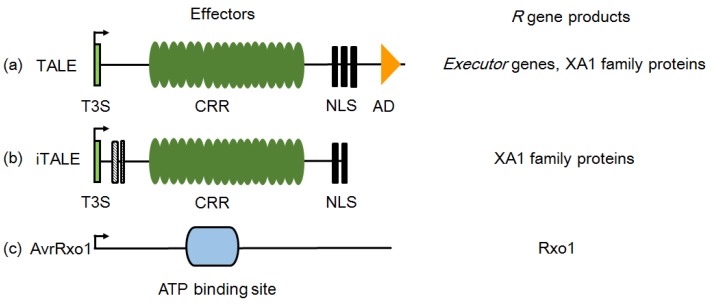
Structural features of identified effector proteins participating in induction or suppression of major *R* gene resistances. (**a**) The typical structure of TALEs. TALEs contain an N-terminal type III secretion and translocation signal (T3S), a specific central repeat region (CRR), three nuclear localization signals (NLS), and a C-terminal transcription activation domain (AD). TALEs play multiple roles in rice–*X. oryzae* interaction and can be recognized by executor and *Xa1* family genes. (**b**) The structure of type-A iTALEs. iTALEs are truncated variants of TALEs. Boxes with stripes represent deletions. In *X*. *oryzae*, iTALEs are prevalent inhibitors of resistance of *Xa1* family genes. The different structures endow TALE and iTALE with opposite functions. (**c**) AvrRxo1 is similar to the zeta toxin protein, and an ATP-binding site exists in the central region. The targeted *R* gene products involved in susceptibility or resistance induced by the effectors are shown on the right.

The nucleotide-binding leucine-rich repeat (NLR) gene family is another big group of *R* genes in plants and the first cloned gene of this type genes conferring resistance against *Xoo* is *Xa1*, which is cloned from rice line IRBB1 [47]. *Xa1* confers high levels of specific resistance to Japanese *Xoo* strain race 1 (T7174), but the mechanism of *Xa1*-mediated resistance is still unclear. A recent study on effects of deleting TALEs in the *Xoo* strain PXO99^A^ revealed that *Xa1* is actually a broad-spectrum resistance gene that can recognize different typical TALEs (tTALEs, identical with TALEs here) and initiate hypersensitive response (HR) cell death [10]. The activation of *Xa1* resistance is independent of the number of TALE’s central repeats (if >3.5 repeats) and the RVDs composition of each repeat. In the natural habitat, such *Xa1* resistance is suppressed by two groups of truncated TALEs (interfering TALEs, iTALEs), e.g., *Tal3a* and *Tal3b* in PXO99^A^. The *Xa1*-mediated resistance is activated by tTALE but suppressed by iTALE independently; both cases are prevalent in *Xoo* and *Xoc* isolates [10,48]. Compared with tTALEs, iTALEs lack 58 amino acids at the N-terminus and the transcriptional activation domain but retain one or two NLSs at the C-terminus, which are conserved in iTALEs and essential for interfering the *Xa1*-mediated resistance (Figure 2 and Figure 3). XA1 possesses a unique nucleotide binding site (NBS) region and its expression is induced by wounding and pathogen infection. The LRR domain located at the carboxy termini is the major diversified region among XA1 family members, and the LRR versions determine the output of resistance. *Xa1* harbors six nearly identical LRR repeats (each repeat with 92 amino acids), but the first two LRR repeats of its susceptible allele (*Os04g53120*) still seem in the course of evolution (Figure 1).

What is more interesting is that T7174, the representative strain of Japanese *Xoo* race 1, shows incompatible interaction with IRBB1 (*Xa1*/*Xa1*), even though it harbors an iTALE (*Tal3b* type). Furthermore, expression of *avrXa7* and *avrXa10* (two tTALEs) or the AD (activation domian)-deleted tTALE *pthXo1CRR* (ΔAD) in rice variety Carolina Gold Select (*Xo1*/*Xo1*) can drastically decrease the virulence of *Xoo* strain X11-5A [31]. These findings suggest that the resistance function of the NLR proteins (here *Xa1* and *Xo1*) may be inhibited by iTALEs. Similar observation has been recently described in a study on the *Phytophthora sojae*–soybean interaction [49]. It appears that iTALEs have been evolved as a paralogous decoy to neutralize the ability of NLR proteins. iTALEs themselves have no function in the development of disease, other than interfering NLR proteins in order to protect tTALE from recognition. *Xa1* is not the sole NLR-type *R* protein that can recognize TALEs or its homologues because *Bs4*, a major *R* gene cloned from tomato, encodes a protein that also recognize the TALE member AvrBs4 from bacterial spot pathogen *Xanthomonas campestris* pv. *vesicatoria* in pepper [50]. The difference between *Bs4* and *Xa1* is that AD domain of elicitor is not required for *Bs4* resistance elicitation as C-terminal-truncated *avrBs4* can also trigger resistance. However, the mechanism of *Bs4* resistance needs to be revealed as well.

## 4. Race-Specific Resistance Mediated by Recessive *R* Genes

TALE proteins were found primarily in plant pathogenic bacteria *Xanthomonas* species, *Ralstonia solanacearum* and *Burkholderia rhizoxinica*. They are special and significant weapons of pathogens in the rice–*X. oryzae* interaction [1]. PthXo1, AvrXa7, PthXo2, PthXo3, TalC, and Tal5 from *Xoo* and Tal2g from *Xoc* are all important virulence determinants, and except Tal2g, all of them target the SWEET family genes [13,14,51,52,53,54]. The first identified *SWEET* gene of TALE target is *Os8N3* (also named as *OsWEET11* and *Xa13*), which encodes a sucrose efflux transporter and can be bound and transcriptionally activated by PthXo1. Deletion of *pthXo1* or silencing of *Os8N3* leads to nearly 80% lesion length reduced upon PXO99^A^ challenge [13]. The induction of *SWEETs* expression might facilitate sucrose movement through intracellular regions of plants, which provides nutrition to support bacterial multiplication and disease development [55]. Some other pathogens, such as *Botrytis cinerea*, also benefit from induction of *SWEET* gene expression during the infection process [56,57,58]. The expression of *Os8N3* is essential for the pathogen but can be exchanged by the other clade III homologs [54]. It is astonishing that no native *SWEET*-targeting TALEs have been identified in *Xoc*, although *SWEET* gene expression induction can benefit *Xoc* infection [59]. *Xoc* genomic research indicates that Tal2g_BLS256_, together with four other TALEs, are highly conserved in *Xoc*. Experiment has shown that Tal2g_BLS256_ can activate a sulfate transporter-encoding gene expression, which is a major susceptible target for BLS. However, there is no explanation on the reason why a TALE contributes greatly for *Xoc* virulence by targeting the *OsSULTR3;6* [52].

To counter the pathogen’s attack, plants mutated the effector binding elements (EBEs) in promoters of the *S* genes to disrupt the TALE-induced *S* target expression. For example, the recessive BB-resistant gene *xa13* evolved from the susceptibility allele *Xa13* by various nucleotide substitution, deletion, and insertion in the EBE region [60]. These nucleotide alterations in the promoter confer race-specific resistance to *Xoo* strains, which apply *pthXo1* as the only major virulence factor, e.g., PXO99^A^. Similarly, *xa25* is another recessive *R* gene and allelic to the *S* gene *OsSWEET13*, which is implicated as a target of major TALEs from *Xoo* strains PXO339 and PXO163 [61]. In addition, *xa41* is an 18bp deletion promoter variant of *OsSWEET14* present only in African rice wild species *O*. *barthii* and *O*. *glaberrima* [32].

An alternative route of rice to counter the action of pathogenic TALEs is to reduce the affinity of TALEs with plant transcription factor. The recessive *xa5* is a natural allele of *Xa5* (TFIIAγ5). TFIIAγ5 is the small (γ) subunit of the conserved general transcription factor TFIIA, which is important for polymerase II(Pol-II)-dependent transcription. The protein encoded by *xa5* contains a mutation in the 39th residue, where the valine (V) residue is replaced with glutamine (E) (V39E). This missense mutation in *xa5* attenuates the transcription of downstream TALE-targeted *S* genes, therefore improving rice resistance by abolishing the interaction between virulence factor TALEs and the preinitiation complex [62]. *OsTFIIAγ1* and *OsTFX1* are two other *S* genes associated with gene transcription; unfortunately, naturally resistant allele has still not been found. Although TFIIAγ1 do not directly interact with TALEs as TFIIAγ5, a recent study displayed that TFIIAγ1 can compensate for the absence of *Xa5* when *Xoo* induces the expression of *OsTFIIAγ1* by PthXo7 [63].

## 5. Race-Specific Resistance Mediated by Executor *R* Genes

In contrast to avoiding damage from pathogen by adopting recessive genes, rice employs another vigorous tactic to defend the fierce attacks from TALEs, which is more aggressive and effective (30). Executor *R* (*E*) genes encode a type of small proteins, conferring dominant resistance to *X. oryzae* and showing no similarity to any other known *R* gene products [30]. The primal TALE’s targets for bacteria are supposed to be some susceptibility (*S*) genes, because induction of *S* genes will benefit bacterial virulence and disease development. *E* genes possess specific EBEs in their promoter regions that can trap the cognate TALE effectors, e.g., AvrBs3 and AvrXa23. *E* genes are silent in normal conditions, but their expression occurs upon recognizing the Avr proteins from pathogen, which triggers immunity response in plants.

Thus far, five *E* genes have been cloned and three of them are from rice, e.g., *Xa10*, *Xa23*, and *Xa27* [64,65,66], whose corresponding *avr* genes are the TALE-encoding genes *avrXa10*, *avrXa23*, and *avrXa27,* respectively. Zhang et al. grouped the E proteins into two groups: Group 1 is likely associated with plant development or physiology and group 2 are small proteins with multiple hydrophobic potential membrane-spanning domain [30]. Bs3, isolated from pepper, is the sole member of group 1, which is structurally related to flavin-dependent monooxygenases of the *Arabidopsis* YUCCA family, and plays roles in auxin biosynthesis and pathogen defense [67]. The *E* genes identified from rice are all classified into group 2. Actually, the *E* gene-related or similar coding sequences, which harbor polymorphism in promoters, are present in different rice cultivars and are mostly similar in function if induced by artificial TALEs. *Xa27* is the first reported *E* gene in rice, encoding a 113-amino-acid protein localized to cytoplasmic membrane and apoplast. Compared to the promoter of susceptible allele *xa27* in rice variety IR24, one GAA trinucleotide repeat insertion and a nucleotide substitution downstream the TATA box endows the resistant allele *Xa27* in IRBB27 with an ability to recognize the natural TALE AvrXa27 [66]. Protein sequence analysis and function studies have indicated that the signal anchor-like sequence at amino (N)-terminal region of XA27 determine the protein localization and is essential for resistance to *Xoo* [68]. The *Xa27* resistance is affected by growth stages and sometimes genetic backgrounds. *Xa10* and *Xa23* reside at two adjacent loci on chromosome 11 and are closely related members, sharing 64% similarity in DNA sequences and 50% identity at protein level [64,69]. Both XA10 and XA23 are endoplasmic reticulum-localized proteins and can induce HR in nonhost plants such as *Nicotiana benthamiana*. XA10 and XA10-like R proteins (e.g., XA10-Ni) have four predicted transmembrane helices, whereas XA23 or XA23-like R proteins have only three predicted transmembrane helices in short N-terminal region [70]. An ED or ED-like motif, comprising acidic amino acids residues (D and E), appears at the C-termini of XA10, XA23, and XA27. This specific domain in XA10 and XA23 has been proven essential for HR induction in tobacco and resistance in rice. All the E proteins appear to be toxic to plant cells, and expression of *E* genes can elicit hypersensitive response (HR) to restrict pathogen growth and diffusion. Further experimentation has revealed that XA10 forms hexamers, then induces ER Ca^2+^ depletion, and triggers cell death in rice, *N. benthamiana*, and human HeLa cells; however, details about how an E protein switches on the HR in host plants is still unclear [65].

Intriguingly, no cognate *S* genes of the TALEs AvrXa10, AvrXa23, and AvrXa27 have been identified. AvrXa27 and AvrXa23 are conserved in various *Xoo* strains; therefore, *Xa27* and *Xa23* have been widely applied in rice breeding programs, and the resulting varieties show broad-spectrum resistance against *Xoo* strains. However, none of the three *E* genes has been utilized to control BLS because natural *Xoc* strains do not carry the cognate TALEs. Additionally, it has been shown that XA10-mediated resistance is sometimes not strong enough to revolt attacks from *Xoc*, even if the *avr* gene *AvrXa10* was introduced into *Xoc* by electrotransformation [71].

## 6. Durable and Broad-Spectrum Resistance Mediated by Nonhost *R* Genes

In contrast to the host disease resistance genes, nonhost resistance genes usually target conserved effectors and show durable and broad-spectrum resistance [72]. *Rxo1* (the same gene as *Rba1*) is a maize NLR-type *R* gene conferring resistance against the maize/sorghum bacterial stripe pathogen *Burkholderia andropogonis* [73]. *Rxo1* conditions a typical HR phenotype in maize and rice upon presence of AvrRxo1 (also termed as XopAJ) or its homolog from *Burkholderia andropogonis* and nonhost bacterial pathogen *Xoc* or *X*. *euvesicatoria* [74]. However, AvrRxo1, a conserved bacterial toxin in Asian *Xoc* strains and some African *Xoc* strains, can enhance *Xoo/Xoc* early proliferation in rice leaves and suppress HR induced by *Xoo/Xoc* in nonhost *N. benthamiana* (Figure 3) [75,76]. The toxicity and virulence contribution of AvrRxo1 might be associated with its nucleotide kinase activity, where AvrRxo1 phosphorylates NAD at the 3′ hydroxyl position to produce 3′-NADP [77]. NAD is a coenzyme and redox carrier that is critically required for organism metabolism function and is an important signal in early events of plant defense responses. AvrRxo1 consumes NAD through direct phosphorylation to manipulate host metabolism and immunity, and the product 3′-NADP could be an inhibitor of defense-related oxidative burst in plants.

The way in which Rxo1 interacts with AvrRxo1 and activates resistance is still a question that needs to be elucidated. A microarray experiment revealed that *Rxo1* functions in the early stage of *Xoc* infection and possibly activates multiple pathways associated with basal defense and HR, such as SA and ET pathways [78]. Besides Rxo1, six *Xop* genes from *Xoc* were transiently expressed and caused HR in *N. benthamiana*, indicating that candidate *R* genes existing in tobacco could be exploited as nonhost *R* genes to improve rice resistance [28].

## 7. Conclusions and Perspectives

The rice–*X. oryzae* pathosystem provides a model system for studying plant–pathogen interactions at a molecular level. Intensive investigations in the past decade have revealed that the resistance and susceptibility of rice to BB and BLS are generally controlled by the molecular interactions between *X. oryzae* effectors and its target genes in rice. Among the 11 rice *R* genes against *X. oryzae* characterized so far, eight (*Xa1*, *xa5*, *Xa10*, *xa13*, *Xa23*, *xa25*, *Xa27*, *xa41*) mediate resistance associated with TALEs from the pathogen; only three (*Xa3*/*Xa26*, *Xa4*, and *Xa21*) encode kinase proteins and mediate rice resistance. This is very special because NLR-type *R* genes are the major players in other plant–pathogen pathosystems, e.g., rice–*M. oryzae* [79]. Moreover, the TALE-associated rice *R* genes against *X. oryzae* play different roles. Based on these advanced information, this review summed up five routes that rice has evolved or has adopted to defend attacks from *X. oryzae*.

Although enormous progress in characterizing rice–*X. oryzae* interaction and breeding for resistance has been achieved since the molecular cloning of the first *R* gene *Xa21* against Xoo, many questions and challenges still remain. For example, nearly a dozen *R* genes against *Xoo* have been cloned, but no effective major *R* gene against *Xoc* has been discovered in rice. In addition, the BB *R* genes cannot be used for BLS resistance in rice breeding, although *Xoo* and *Xoc* are close species and even possess similar biological weapons, e.g., Xop proteins and TALEs. The lack of endogenous BLS *R* gene can be partially explained for immense and expansionary TALomes of *Xoc*. The *Xoc* strains may target diverse *S* genes to exploit nutrients, which is different from other *Xanthomonas* species. Another factor is that *Xoc* TALEs function to suppress multiple resistance, for example, both AvrXa7 and Tal2a_BLS256_ with *Xoc* background failed to initiate expected HR in the corresponding resistant rice [16,80]. Elucidation of the function mechanism of TALE-like proteins (Tal7_RS105_ and Tal3a_PXO99A_) exerting immunity suppressors is the basis to engineer effective *R* genes against *Xoc* and also *Xoo*. Up to now, the genetic working model of executor *R* genes has been well addressed. However, many biochemical details about how an E protein switches on the HR in host cells is still to be elucidated, even though there is a clue associated with induced ER Ca^2+^ depletion [65]. Elucidation of the detailed biochemical mechanism of the executor *R* genes is a great challenge because the cell death is too fast to track the biochemical process easily.

The *Xoo* and *Xoc* populations exhibit high genetic diversity and present rapid evolution in natural environments. New aggressive strains/races have often emerged, eroded the *R* gene resistance, and expanded in different regions, probably due to strong host selection, changing ecological conditions, and farm managements [81]. In this scenario, natural genetic resources may not be enough to continuously breed new, resistant rice cultivars in the future. TALENs (transcription activator-like effector nucleases) and CRISPR (clustered regularly interspaced short palindromic repeats)/Cas9 (CRISPR-associated 9) system-based genome editing technologies have become essential to develop new crop varieties with durable and broad-spectrum resistance. To this aim, *Os11N3* (*OsSWEET14*), the *S* gene targeted by AvrXa7 and PthXo3, has been edited by TALENs to create BB-resistant rice through disrupting the EBE site in the promoter region [82,83]. Moreover, CRISPR/Cas9 is a new, simple, and more effective tool for genome editing system, which even enables multiplex gene editing in many organisms, including rice [84,85]. Two recent reports have provided successful examples of enhancing rice blast resistance via CRISPR/Cas9-mediated knock-out of *OsERF922* and *Bsr-d1* [86,87]. It appears that the CRISPR/Cas9 will become an efficient and frequently used technology, accelerating both basic and applied research on the rice–*X. oryzae* pathosystem. Except for genome editing, artificial *E* genes with designed EBE could become another effective approach to achieve plant resistance because a designed EBE can trap multiple or widely conserved TALEs that enable the artificial *E* gene to resist *X. oryzae* strains broadly [88,89].
